# Mapping the distribution of specific antibody interaction forces on individual red blood cells

**DOI:** 10.1038/srep41956

**Published:** 2017-02-03

**Authors:** Natasha Yeow, Rico F. Tabor, Gil Garnier

**Affiliations:** 1 Bioresource Processing Research Institute Australia (BioPRIA), Department of Chemical Engineering, Monash University, Clayton, VIC 3800, Australia; 2 School of Chemistry, Monash University, Clayton, VIC 3800, Australia

## Abstract

Current blood typing methods rely on the agglutination of red blood cells (RBCs) to macroscopically indicate a positive result. An indirect agglutination mechanism is required when blood typing with IgG forms of antibodies. To date, the interaction forces between anti-IgG and IgG antibodies have been poorly quantified, and blood group related antigens have never been quantified with the atomic force microscope (AFM). Instead, the total intensity resulting from fluorescent-tagged antibodies adsorbed on RBC has been measured to calculate an average antigen density on a series of RBCs. In this study we mapped specific antibody interaction forces on the RBC surface. AFM cantilever tips functionalized with anti-IgG were used to probe RBCs incubated with specific IgG antibodies. This work provides unique insight into antibody-antigen interactions in their native cell-bound location, and crucially, on a per-cell basis rather than an ensemble average set of properties. Force profiles obtained from the AFM directly provide not only the anti-IgG – IgG antibody interaction force, but also the spatial distribution and density of antigens over a single cell. This new understanding might be translated into the development of very selective and quantitative interactions that underpin the action of drugs in the treatment of frontier illnesses.

The vast majority of blood typing methods and the totality of testing for blood banking and transfusion are based on agglutination to determine blood compatibility. A known antibody is mixed with red blood cells (RBCs) of an unknown group to type or vice versa^1^. Agglutination occurs when the antibodies recognize and bind to specific target antigens on the RBC surface, subsequently linking multiple RBCs together to form an agglutinate in a positive reaction^2^. RBCs are weakly electrostatically stabilized under normal physiological conditions with a moderate negative charge (ς = −15 mV)^3^. During blood typing, the pentameric IgM is able to directly agglutinate IgM-sensitized RBCs while a bridging element, usually anti-IgG, is required to agglutinate IgG-sensitized RBCs (Fig. 1)^2^.

Zhang *et al*. first imaged fine structures on the red blood cell in air using the atomic force microscope (AFM). The surface of a RBC was divided into 28 sub-areas to map the whole surface of the RBC at a nanometer resolution. Images of sub-areas were assembled to reveal the fine structures on the entire cell surface. The specific components observed in their study may be associated with different proteins on the surface of the RBC membrane^7^, but the functions of those fine structures were not identified in the study. Touhami *et al*. functionalized the AFM cantilever with anti-A antibodies to measure antigen-antibody interactions on a RBC^8^ but no supporting results were shown. Grandbois *et al*. managed to distinguish 2 different groups of RBCs in a mixed pool of cells using the AFM with a combination of AFM tip functionalized with *Helix pomatia* lectin^9^. None of the AFM work to date has studied the direct interaction of blood grouping antibodies with blood group-associated surface antigens on the RBC.

Surface Plasmon Resonance (SPR) has been investigated to quantify the kinetics of antibody-antigen interactions. The SPR is able to detect the presence of antibody-antigen interactions by the increase of response units but does not provide direct measurement of the binding energy involved in the interactions^10^,^11^,^12^. No binding energy between blood grouping antibodies and RBC surface antigens has been reported. A recent study reported the use of SPR to perform quantitative blood typing based on the indirect antiglobulin test, where anti-IgG antibodies are used to identify IgG-sensitised RBCs^13^. Likewise, the interaction force between the anti-IgG and IgG antibodies on red blood cells was not measured. Our study exploits the ability of the AFM to measure the interaction force between anti-IgG and IgG bound to RBC surface antigens. 2D mapping of a series of surface force measurements scanned on a single RBC at different intervals allowed us to quantify the antigen site density and localize the antigen sites on the surface of the RBC membrane. RBC antigen density was previously measured by flow cytometry techniques where RBCs were stained by fluorescent-tagged antibodies^14^.

Here, for the first time, we map the distribution of antigens directly on single RBCs by measuring the antigen-antibody interaction force with AFM by scanning a series of individual RBCs. In our method, the tip of an AFM is functionalized with an antibody (anti-IgG); RBCs are incubated with a selective or with a non-selective antibody (IgG). A series of RBC are scanned over individually by AFM with a cantilever tip functionalized with anti-IgG that recognizes and reacts only with IgG antibodies adsorbed on a selective RBC surface antigen. A force curve is made at each contact point, providing in depth statistics of the interaction forces at various length scales and locations on the curved RBC.

## Results and Discussion

**Figure 2:**
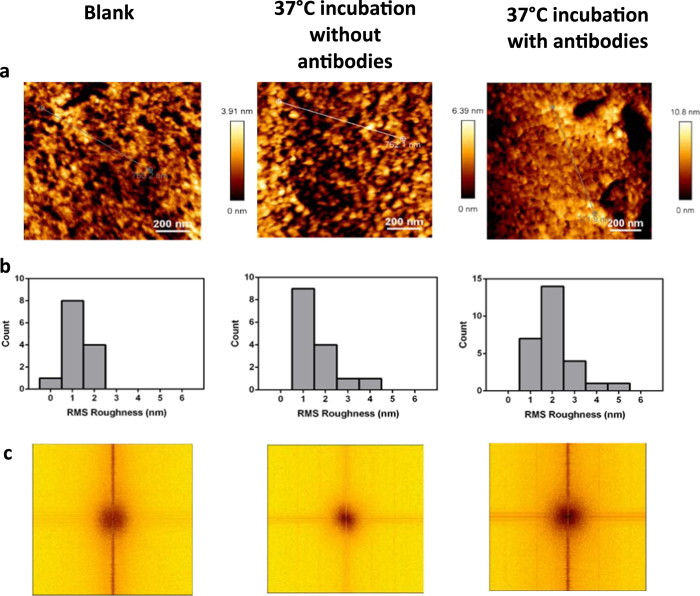
RBC imaging in air. (**a**) AFM images (1 μm × 1 μm) of RBC surface for blank, RBC incubated at 37 °C without antibodies and RBC incubated at 37 °C with selective antibodies. (**b**) Histograms of RMS roughness for different samples. (**c**) Fourier transform analysis for the different samples.

The surface roughness study was performed to locate the IgG antibodies bound to surface antigens. Red blood cells of blood groups D+ and D−, and the anti-D in IgG form, were used as representative model for our study. A surface roughness investigation on D+ RBCs incubated with its specific antibodies (anti-D) against blank and temperature controls showed that D+ RBC samples incubated with IgG anti-D antibodies had the highest root mean square (RMS) roughness (Fig. 2a,b). Incubating RBCs at 37 °C increased the surface roughness though not significantly. However, features at a greater height were only observed for the positive samples and not in the controls. We interpreted that both phenomena were somehow linked to the presence of IgG antibodies on the RBC surface. (Table 1, Fig. 2b). The average feature height measured ranges from 4.7 nm to 6.3 nm. For comparison, the dimension of the IgG antibody measured using cryo-AFM is approximately 12 nm (length) × 12 nm (width) × 3 nm (height)^6^. The difference between the mean roughness of RBCs incubated at 37 °C and the height of the tall features found in both samples does not correlate well with the dimensions of IgG antibodies. This could be due to a change in conformation of the antibody during the adsorption onto antigen sites at an angle. Spatial (2D) Fourier transformations provide information on whether certain spatial frequencies are repeated within the image, hence giving insight as to whether there is regular spacing between surface topological features. A strong characteristic set of lines would be evident in this circumstance. However, this was not observed in the transformations. Instead, the weak spatial signatures in the Fourier transform analysis of the surface topology of the 3 samples showed some evidence of repeating size motifs, but no obvious periodicity, suggesting that the features, which are possibly IgG antibodies bound to antigen sites, are similarly sized but located randomly on the surface of the RBC (Fig. 2c). As RBCs are very soft biological matter, imaging the cell surface in liquid without chemical fixation might not produce clear enough definition to identify the presence of IgG on the cell surface. Imaging the intact RBC in air resulted in higher resolution of the surface topography. However, due to the presence of nanostructures on the RBC surface, it was difficult to identify IgG antibodies. We were only able to identify repeating size motifs but unable to confirm that they are the antibodies. Thus, imaging alone provides an incomplete understanding of the location and distribution of antibodies on the cell surface, and motivates an investigation of specific binding forces to better map the antibody locations.

**Figure 3:**
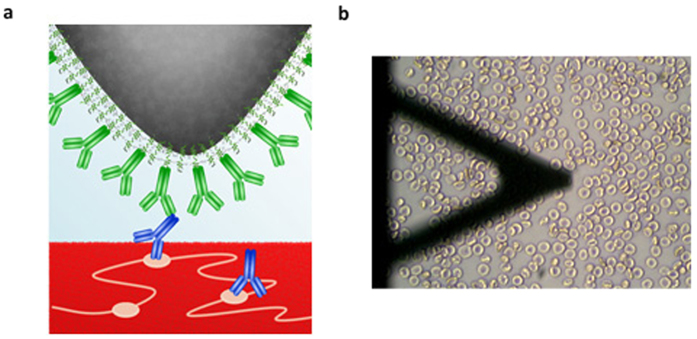
Force mapping in liquid. (**a**) 3D model of AFM force work. (**b**) AFM cantilever tip functionalized with anti-IgG with RBCs attached to poly-L-lysine treated Petri dish surface.

To identify the features responsible for the increase in surface roughness of RBCs incubated at 37 °C with specific IgG antibodies, we functionalized AFM cantilevers with anti-IgG, a molecule able to selectively link with the Fc region of the IgG antibodies bound to the RBC antigens after incubation (Fig. 3a). Force measurement in air was inconclusive; this was likely due to the interference created by water capillary bridging between the functionalized cantilever and the RBC sample surface.

**Figure 4:**
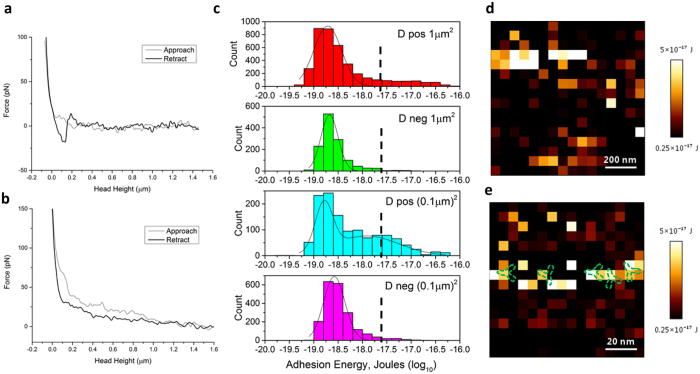
Experimental results for force mapping. (**a**) Typical force curve of a single specific interaction and (**b**) a non-interacting retraction. (**c**) Histograms of adhesion energies recorded from 1 μm^2^ and (0.1 μm)^2^ scan areas for D positive and negative samples. Black dotted lines represent the minimum threshold (0.25 × 10^−17^ J) for specific interactions. (**d**–**e**) Images showing a range of interaction energies (depicted by the colour scale) higher than 0.25 × 10^−17^ J on different points of the scan area. The typical size and potential conformation of IgG antibodies (green dotted lines) bound to the specific antigens is shown in (**e**).

The AFM technique was modified to directly scan RBCs in liquid. RBC samples (D+, D−) incubated with anti-D antibodies and blank RBCs (no incubation) were bound to a poly-L-lysine treated glass surface and submerged in a cell preservative solution. Force mapping was used to detect and measure interaction forces between anti-IgG on the cantilever and IgG antibodies on the RBC surface- if present. Utilizing the inverted optical microscope on the AFM setup, we were able to single out individual RBCs for force mapping (Fig. 3b). Total scan areas of 1 μm^2^ and (0.1 μm)^2^ were utilized on the surface of a series of red blood cells with 16 × 16 pixels, thus avoiding the collection of data from non-specific adhesions between the functionalized cantilever and the Petri dish surface. In the force-mapping mode, each pixel represents the functionalized cantilever approaching the sample surface and then producing an individual force-distance interaction curve (Fig. 4a,b, Supplementary Information Figure S1a–c). Adhesion energies are obtained by integrating the area under the retract branch of each force curve, and binned into histograms as shown in Fig. 4c. From both 1 μm^2^ and (0.1 μm)^2^ scans on the red blood cell surface, it became clear that higher interaction forces were only recorded for the positive samples; these are seen in the tailing on the right hand side of Fig. 4c. Higher interaction forces are more apparent in (0.1 μm)^2^ compared to 1 μm^2^ scans. This may be due to the distance between each measurement (in pixel) on the force mapping, namely 62.5 nm for 1 μm^2^ scan areas versus 6.25 nm for (0.1 μm)^2^ scans. Based on the dimensions of the IgG antibodies (12 nm × 12 nm × 3 nm), there is a high probability that the cantilever is coming into contact with the specific interacting Fc region of an IgG antibody multiple times in the (0.1 μm)^2^ scan. Meanwhile, it is assumed that each specific interaction recorded for 1 μm^2^ scans reflects a specific contact with a single antibody due to the much larger distance between each scan. This further suggests that analysis of antigen density and distribution should be established from 1 μm^2^ scans for higher accuracy. Data from (0.1 μm)^2^ could provide information on the localization of the IgG antibody bound to RBC surface antigens. The other low or near zero adhesion energies measured can be attributed to the cantilever coming into contact with the surface of RBC where no IgG is present or to interactions with non-specific areas of the IgG antibodies. Similar typical force curves showing specific interaction and no interaction between antibodies functionalized on AFM tips and the receptor on the substrate surface or vice versa were also observed in other AFM studies on antibody-antigen interactions^15^,^16^. Therefore, the force measurement performed for positives measures two additive contributions; a specific contribution, and a non-specific contribution. The specific contribution corresponds to the interaction force between anti-IgG on the cantilever tip and the IgG bonded to its selective RBC antigen; this component is only for bio-specific interactions. The non-specific force contribution is made of generic electrostatic, van der Waals and hydrophobic forces; it is present for both negatives and positives. Although a force of a few tens of pN is sufficient to remove the lipid moiety of a receptor from the cell membrane, the possibility of the antigen being removed during the probing of the cantilever tip is unlikely in our case as the transmembrane RhD antigen is part of a membrane complex connected to the spectrin matrix of the cell membrane cytoskeleton^9^,^17^. Additional controls were performed to demonstrate specific interactions between anti-IgG and IgG bound to RBC surface antigens (Supplementary Information Figure S2a,b).

The minimum threshold for adhesion energies we attribute to specific interactions between the anti-IgG on the cantilever and the IgG antibodies on the RBC surface was set at 0.25 × 10^−17 ^J based on the statistical analysis of our results; no information is available from literature. The histograms of positive samples with different scan areas showed an apparent skew to the right (higher energy values) with the (0.1 μm)^2^ scans displaying an obvious second peak. The minimum threshold value was not set at the centre of the second peak but rather skewed to the right of the peak as there was an evident cut-off at 0.25 × 10^−17 ^J. Interaction energies recorded above the minimum threshold was significantly lower with the negative samples, which saw a percentage of 2.83% and 1.56% for scan areas of (0.1 μm)^2^ and 1 μm^2^, respectively. This is in comparison to the 19.01% and 12.62% for positive samples at scan areas of (0.1 μm)^2^ and 1 μm^2^. Figure 4(d,e) presents the images mapping the adhesion energies above the threshold (0.25 × 10^−17 ^J) achieved by random scanning areas on the surface of a series of RBCs incubated with its specific IgG antibody. The range of adhesion energies is depicted using a color scale for clarity. Force maps of negative controls showing no specific interactions occurring between the functionalized cantilever and D negative cells are shown in Supplementary Information Fig. S3. According to 1 μm^2^ scans, points of adhesion energies above the threshold are randomly scattered. Analysis over a few samples showed no regular pattern of interaction sites. This observation is in agreement with the previous Fourier Transform analysis for the surface roughness study, where no regular pattern was observed for the spatial features interpreted as IgG antibodies. As there is no way to measure where on the IgG antibody did the anti-IgG come into contact, we can only interpret the specific interactions as a range of value with 0.25 × 10^−17 ^J being the minimum binding energy. The maximum binding energy recorded was 5.92 × 10^−17 ^J and 8.19 × 10^−17 ^J for (0.1 μm)^2^ and 1 μm^2^ scans, respectively. As discussed earlier, (0.1 μm)^2^ scans are likely to over-report antigen density due to the potential for multiple interactions with a single antigen. However, the distribution of adhesion energies within these scans facilitate estimation of the IgG antibody conformation when bound to surface antigens. Figure 4e shows specific interaction forces being cluttered in a non-random fashion. A prediction of the IgG antibody localization on the cell surface was made based on the clusters of specific interactions. The exact orientation of the IgG antibodies were not directly measured.

Statistical analysis of the interaction forces from the force mapping work also provided information on the distribution of the IgG anti-D antibodies on the RBC surface, indirectly representing the distribution of D antigens. The unknown conformation of IgG antibodies bound to RBC surface antigens render interpretation of the results challenging. The D antigen (RhD) has multiple epitopes on the extracellular membrane. Based on the relative mass of the RhD protein (31.9 kDa)^18^ and the molecular weight of IgG molecule (150 kDa)^19^, we assume that only one IgG anti-D molecule is able to bind to one epitope which blocks the other epitopes. Therefore one anti-D recorded equals to one antigen site. Each interaction over 0.25 × 10^−17 ^J is assumed to be an adhesion event between the anti-IgG on the cantilever and the IgG anti-D bound to a D antigen on the RBC surface. Based on this assumption, the average separation (2*r*) found between IgG anti-D binding sites in 1 μm^2^ scans ranged from 0.109 to 1.151 μm; *r* being the radius of the area of each individual interaction recorded. The density of D antigens quantitated from 1 μm^2^ scan area ranged from 2 to 182. By assuming that the surface area of an adult RBC is 134.3 μm^2^ 20, the average RhD antigen density per cell on a RBC surface ranged from 252 to 23,168 sites per cell (Table 2). The results appear to fall in the range of the reported distribution of RhD antigens on a normal RBC which is 10,000–33,000^21^. When estimating the antigen density per cell from 1 μm^2^ scan area images, it appears that some scanned areas exhibits antigen sites below the reported range, suggesting that the D antigen is not uniformly distributed on the RBC membrane surface. This has been reported by earlier studies using anti-human gamma globulin conjugated with ferritin and gold labelling with electron microscopy^22^,^23^. Therefore, the assumption of antigen sites per cell based on small individual scan areas is not sufficient. A series of scans of the whole cell and the combination of antigen densities obtained from each individual scan will provide a more accurate quantification of antigen sites. Table 2 shows that no consistency was observed in the distribution of antigens within cells of a single individual. This could be due to the non-uniform distribution of the antigens. When areas on the cell were randomly selected for force mapping, varying amounts of antigens are identified in that particular mapped area, resulting in a different total antigen density per cell. This also applies to the heterogeneity of D antigen density observed for cells among individuals. Indeed, this heterogeneity has been previously reported by Hughes-Jones *et al*.^24^.

In this study, we developed a novel method to map the distribution of antigens on single RBCs. First, we analyzed the surface roughness of a series of RBC in air with the tapping mode. We then mapped the interaction forces between anti-IgG antibody and the IgG antibody bound to the surface of individual RBCs in liquid at different length scales. The force mapping in liquid can be applied in further investigation of RBC surface antigens as a function of varying physiological conditions, e.g. change in pH, ionic strength and temperature, etc. The AFM also allows isolation of a single cell to be studied. Mapping specific biological interactions *in situ* and on a per cell basis can provide deep insight into biochemical processes and the role of cellular machinery which is not delivered by other blood typing methods. Quantifying the antigen density and the antibody-antigen force on RBC open new avenues of very selective and precise drug delivery such as for blood cancer treatments. Furthermore, the new concepts developed also enable for the first time to measure ligand-receptor heterogeneity among the cells of an individual, or among individuals in a population, therefore facilitating precise personal treatment.

## Methods

### Red blood cell surface imaging in air

Human RBCs from different sources (AbtectcellTM III 3%, Commonwealth Serum Laboratory (CSL) and donor samples obtained from Australian Red Cross Blood Service diluted to 3% in ID CellStab, Bio-Rad Laboratories Pty. Ltd.) were prepared for 3 different conditions. 1) Blank controls were RBCs without any form of incubation. 2) RBCs for temperature control were incubated at 37 °C for 30 minutes without addition of antibodies. 3) RBCs for positive samples were D+ incubated at 37 °C for 30 minutes with 2% for further manufacturing use (FFMU) IgG anti-D for further manufacturing use (FFMU), CSL. Blood smears from the 3 samples were prepared on microscope glass slides and air dried. Glass slides were imaged with a JPK Nanowizard 3 AFM in AC (intermittent contact) mode using Bruker NCHV model cantilevers, which had nominal resonant frequencies of 320 kHz and spring constants of 42 N/m. Images were obtained with a set-point force of <1 nN. Scans of 1 μm × 1 μm on random regions of the red blood cell surface were performed for the RMS roughness study.

### Cantilever functionalization

Cantilevers used in the force mapping work are the Bruker MSCT model cantilevers. The Hutter and Bechhoefer method^25^ was utilized to determine the spring constants of each cantilever; they ranged from 0.0378 to 0.2433 N/m. Cantilevers were functionalized using a protocol developed by Nugaeva *et al*.^26^. Silicon nitride cantilevers were silanized, activated with glutaraldehyde and treated with staphylococcal protein A (Calbiochem). Finally, the cantilevers were functionalized with anti-human IgG (Alba Bioscience, UK) adjusted to 500 μg/ml.

### Force mapping in liquid

Human RBC samples were prepared again in 3 different conditions. (1) Blank controls were RBCs in its original state without any incubation. (2) Negative samples were known D- RBCs incubated with 2% IgG anti-D at 37 °C for 30 minutes. (3) Positive samples were known D+ RBCs incubated with 2% IgG anti-D at 37 °C for 30 minutes. Glass Petri dishes were pre-treated with Poly-L-Lysine 0.01% solution, mol wt 70,000–150,000 (Sigma-Aldrich, USA) in accordance with the Poly-L-Lysine Cell Attachment Protocol provided by the manufacturer (George Sitterley, *Biofiles 2008, 3.8, 12*.). The different RBC samples are left to attach on the Poly-L-Lysine treated glass petri dish submerged in phosphate buffered saline (Sigma-Aldrich, USA) for 10 minutes and washed gently with PBS to remove loose unattached cells. RBCs attached to the glass Petri dish were then resubmerged in Celpresol (CSL, Australia).

Force mapping studies were conducted using a JPK Nanowizard 3 AFM with the contact mode force mapping option. Scan areas of 1 μm × 1 μm and 0.1 μm × 0.1 μm on random areas of the RBC surface were conducted to collect interaction force data between the anti-IgG on the cantilever and the IgG antibody, if present on cell surface. More than 3 cells for each sample was scanned and each cell were scanned for more than 3 times. Each scan was performed with a resolution of 16 × 16 pixels.

### Statistical Analysis

At least 3 cells were scanned for each sample with each cell being scanned at least 3 times. Statistical analysis was performed using the OriginPro 9.1 software.

## Additional Information

**How to cite this article**: Yeow, N. *et al*. Mapping the distribution of specific antibody interaction forces on individual red blood cells. *Sci. Rep.* **7**, 41956; doi: 10.1038/srep41956 (2017).

**Publisher's note:** Springer Nature remains neutral with regard to jurisdictional claims in published maps and institutional affiliations.

## Supplementary Material

Supplementary Information

## Figures and Tables

**Figure 1:**
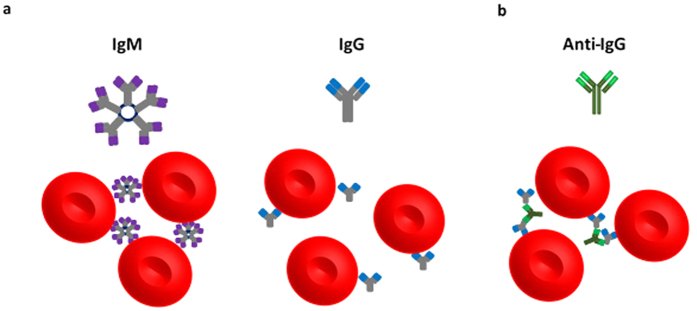
Schematic representation of the blood typing antibody system and the red blood cells (~8 μm)^4^. (**a**) IgM has a large pentameric structure (35 nm)^5^ with 10 antigen binding sites and is active at room temperature while IgG has a monomeric structure (12 nm)^6^ with only 2 antigen binding sites and is active at body temperature (37 °C). IgM is able to directly agglutinate IgM-sensitized red blood cells. IgG antibodies require a bridging element, anti-IgG in this study, to form agglutinates of IgG-sensitized red blood cells. (**b**) Anti-IgG binds to the Fc region of IgG antibodies, enabling agglutination of IgG-sensitized red blood cells. Figures are not to scale.

**Table 1:** Effect of specific antibody binding on red blood cell roughness as measured by AFM on a series of individual cells.

Samples	Mean RMS Roughness (nm)	Features (nm)
Blank (no incubation)	1.17 ± 0.45	≤4.7
37 °C incubation without antibodies	1.63 ± 0.78	≤5.7
37 ° incubation with specific antibodies	2.17 ± 0.96	≥6.3

Features refer to the height profile.

**Table 2:** Antigen distribution per cell quantitated by AFM for positive cells from 1 μm^2^ scans.

Individual	Antigen Density per cell		
	*Based on cell area of 134.3 μm^2^		
	A	B	C
Cell 1	4567	3052	1003
	2497	252	465
	1577	1020	10514
Cell 2	4414	1621	9612
	2136	18923	3235
	23168	18791	2297
