# Supplementary material for: Mapping the distribution of specific antibody interaction forces on individual red blood cells
Source: Sci Rep. 2017 Feb 3;7:41956. doi: 10.1038/srep41956 (PMC5291206; doi:10.1038/srep41956)
Supplement: Supplementary Information [file srep41956-s1.pdf]

# Mapping the distribution of specific antibody interaction forces on individual red blood cells

Natasha Yeow[1], Rico F. Tabor[2*] and Gil Garnier[1*]

[1] Bioresource Processing Research Institute Australia (BioPRIA), Department of Chemical Engineering, Monash University, Clayton, VIC 3800, Australia

[2] School of Chemistry, Monash University, Clayton, VIC 3800, Australia.

*Rico F. Tabor (rico.tabor@monash.edu)

*Gil Garnier (gil.garnier@monash.edu)

## Supplementary Information

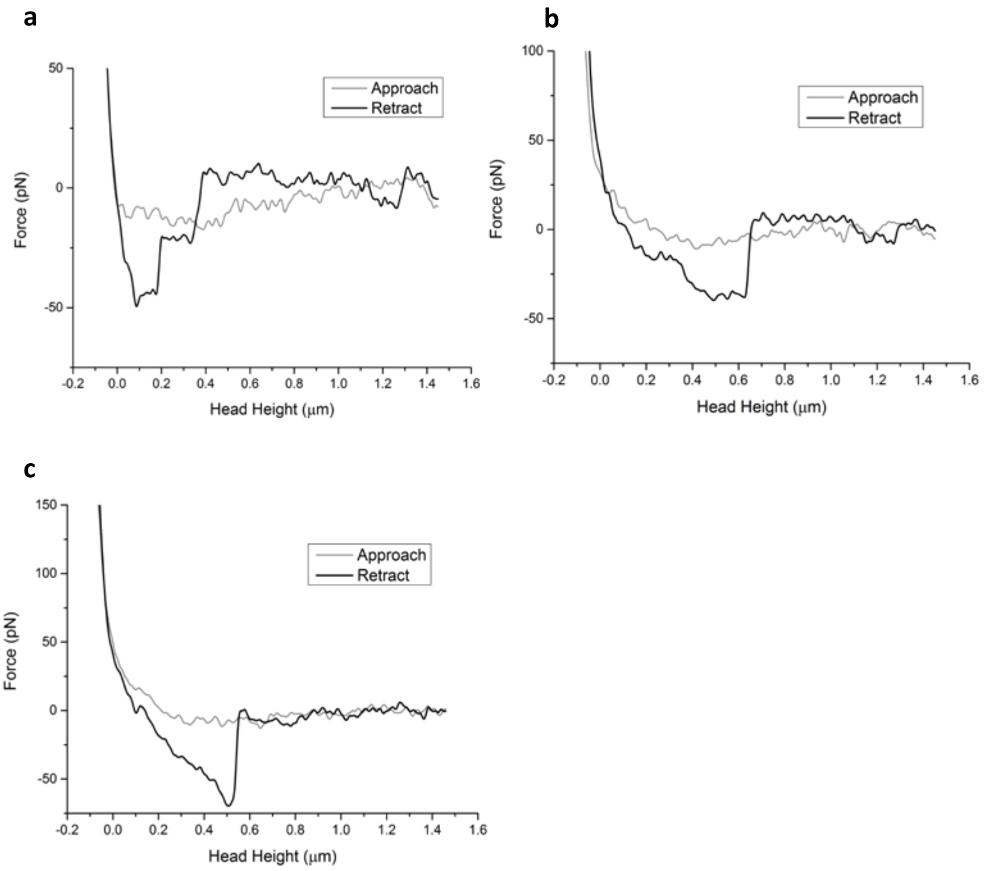


**Supplementary Figure S1.** Typical examples of force curves representing interactions between the anti-IgG functionalized cantilever and IgG antibodies bound to RBC surface antigens on the RBC surface. We categorise the adhesive behaviour that we assign to specific interactions into three broad classes. **a,** step-like detachment, with two distinct force plateaus,

the first of which is largest in magnitude. This most likely represents either two interaction events (spatially unlikely) or a detachment that proceeds via two stages. Given the bulk and complexity of the biomolecules involved, this is not surprising. **b**, similar features as **a**, but with the stronger interaction occurring second, again indicating stepwise separation of the functionalised cantilever and surface. **c**, a single, strong detachment event. The minimum force at disconnection in each case is rather consistent at ~50 pN, indicating that the specific peak interaction force is independent of the detachment mechanism. As antibody-antigen interactions are known to be both spatially and orientationally specific, we would expect to sample a range of different specific force profiles depending on the specific orientations of the antibody and antigen involved in each interaction pair.

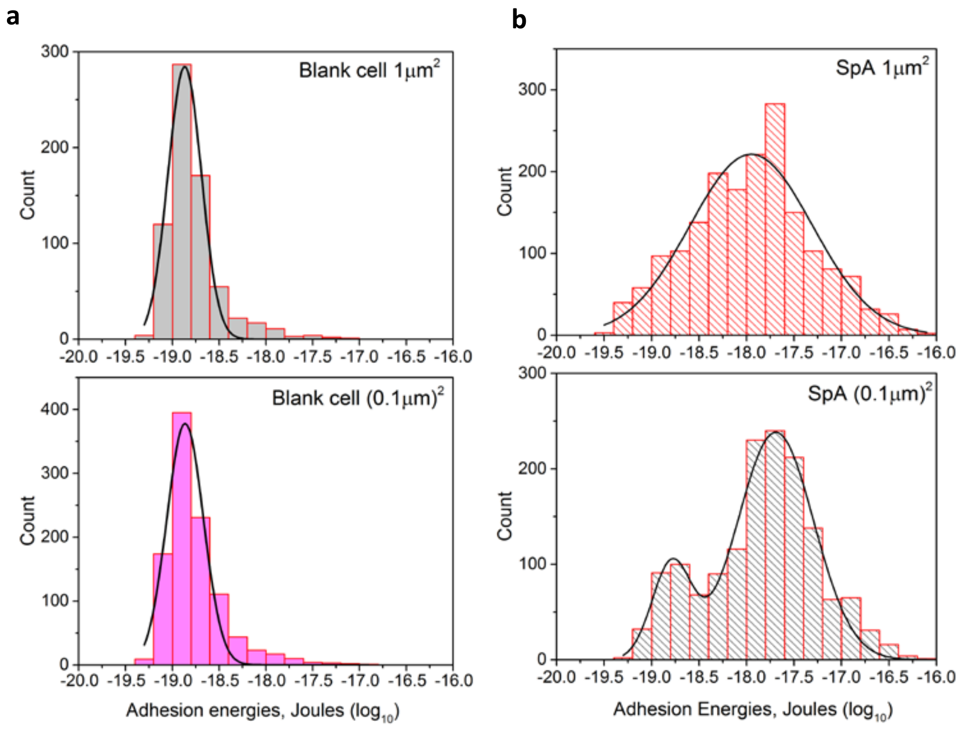


**Supplementary Figure S2.** Histograms for force mapping scans on **a,** blank cells; the count frequency at each level of binding adhesion between the functionalized AFM tip and red blood cells without incubation. **b,** D positive cells with cantilevers pre-functionalized with Staphylococcal protein A (SpA). The probability of protein A interacting with RBC surface antigen bound IgG antibodies appears to be higher than that of anti-IgG interacting with the IgG antibodies. This may be due to the number of IgG binding sites on protein A, which is five binding sites[1] against two on an anti-IgG antibody. However, it is believed that only one anti-IgG molecule (150 kDa) is able to bind to one SpA molecule (42 kDa).[2] Therefore, non-specific interactions between SpA on the cantilever and IgG anti-D antibodies on the RBC surface has been minimized by a saturation of anti-IgG antibodies (5×) in the final cantilever functionalization step.

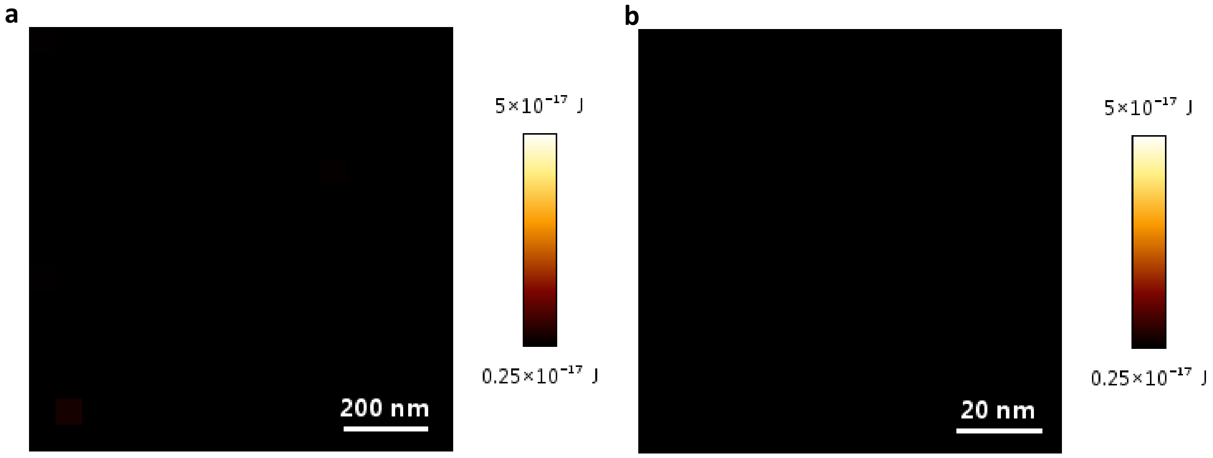


**Supplementary Figure S3.** Force maps of negative controls showing no specific interactions have occurred between anti-IgG-functionalized cantilevers and D negative cells. **a,** Scan area of 1 µm$^2$. **b,** Scan area of (0.1 µm)$^2$.

**References:**

1 Moks, T. *et al.* Staphylococcal protein A consists of five IgG-binding domains. *European Journal of Biochemistry* **156**, 637-643 (1986).

2 Björk, I., Petersson, B.-Å. & Sjöquist, J. Some Physicochemical Properties of Protein A from Staphylococcus aureus. *European Journal of Biochemistry* **29**, 579-584, doi:10.1111/j.1432-1033.1972.tb02024.x (1972).
